# The Anti-diarrheal Activity of the Non-toxic Dihuang Powder in Mice

**DOI:** 10.3389/fphar.2018.01037

**Published:** 2018-09-13

**Authors:** Xiaofei Shang, Xiaolou Miao, Feng Yang, Bing Li, Xiao Guo, Hu Pan, Yu Zhang, Jiyu Zhang

**Affiliations:** ^1^Key Laboratory of New Animal Drug Project of Gansu Province, Key Laboratory of Veterinary Pharmaceutical Development, Ministry of Agriculture, Lanzhou Institute of Husbandry and Pharmaceutical Sciences of Chinese Academy of Agricultural Sciences, Lanzhou, China; ^2^Tibetan Medicine Research Center of Qinghai University, Qinghai University Tibetan Medical College, Qinghai University, Xining, China; ^3^Department of Emergency, Lanzhou Army General Hospital, Lanzhou, China

**Keywords:** Dihuang powder, quality control, toxicity, anti-diarrheal activity, anti-inflammatory activity

## Abstract

Dihuang powder (DHP) has been used in the traditional Chinese medicine for the treatment of diarrhea in some regions of China. But up to now, the anti-diarrheal activity of DHP haven’t been performed with modern pharmacological technology. This study aims to investigate the quality control, the potential toxicity and anti-diarrheal activity of Dihuang powder in mice. High performance liquid chromatography (HPLC) and thin layer chromatography (TLC) were used to detect five active compounds in DHP for quality control, and the acute toxicity and sub-acute toxicity for 28-day oral administration of DHP were then evaluated. The anti-diarrheal activity was investigated using mouse model. Results showed that the levels of quercetin and berberine in DHP were 0.054 and 0.632 mg/g, respectively, and atractylodin, matrine, and patehouli aleohal were also detected in DHP. At the given doses, DHP was safe in terms of acute and sub-acute toxicity. Meanwhile, DHP exhibited strong anti-diarrheal effects as well as decreased gastrointestinal motility and the secretions induced by Sennae and castor oil in a dose-dependent manner. It could decrease the content of IL-1β, IL-6, and TNF-α in the small intestine, and improve the histopathological changes of small intestine and large intestine induced by Sennae. The antinociceptive and anti-inflammatory activities *in vivo* also were presented. Based on all of the results, we thought that DHP has anti-diarrheal activity, and could be used to treat diarrhea as well as alleviate the pain and inflammation induced by diarrhea. This study provides a theoretical basis for the clinical use of DHP and may assist in the development of new drugs for the treatment of diarrhea. The mechanism of the anti-diarrheal activity should be investigated in the future.

## Introduction

Diarrhea, a common gastrointestinal disorder characterized by an increased frequency of bowel movements, wet stool and abdominal pains, is a serious public health challenge (Ezekwesili et al., 2004; Wang et al., 2015). According to the World Health Organization (WHO), an estimated 3–5 billion diarrhea cases occur every year and approximately 5 million people die from diarrhea annually worldwide. Most cases occur in developing countries, especially in the African and South-East Asian regions (Venkatesan et al., 2005; Gutierrez et al., 2008; Page et al., 2011; Tadesse et al., 2017). Diarrhea can be triggered by many different factors, such as infections, food intolerance, intestinal disorders, among others (Young and Schmidt, 2004; Pimentel et al., 2010; Al Jarousha et al., 2011; Barnoy et al., 2011), and also occurs as a symptom of many other diseases, including diabetes mellitus, inflammatory bowel disease, etc. (Farthing, 2000; Benson et al., 2004; Semenya and Maroyi, 2012).

Currently, the treatment for diarrhea is non-specific and is usually aimed at reducing the discomfort and inconvenience of frequent bowel movements (Brunton, 2008; Suleiman et al., 2008). Chemical drugs are often used to treat diarrhea, but adverse effects can be induced by these drugs (Semenya and Maroyi, 2012). To avoid diarrhea, doctors, and patients from many developing countries, such as China, still rely on traditional medicines (Heinrich et al., 2005). For this reason, the WHO has introduced a program for diarrheal control that involves the use of traditional herbal medicines (Agunu et al., 2005).

Traditional Chinese medicine (TCM) has been confirmed to be effective in the treatment of diarrhea in both humans and animals after use in clinical practice for thousands of years, and many TCMs are effective at controlling the development of this disease (Guo et al., 2014; Wang et al., 2015; Yao et al., 2017). As one of the well-known TCM preparations used in the Gansu province of China, Dihuang powder (DHP), was modified from the folk prescription ‘Huopo Heji’ based on TCM theory and has been shown to be effective in the clinic and is widely used to treat diarrhea (Wang and Yan, 1983; Xia and Shi, 2006; Shang et al., 2015a). It consists of six herbal medicines, including Euphorbiae Humifusae Herba (from the whole plant *Euphorbia humifusa* Willd. or *E. Maculata* L.), Coptidis Rhizoma (from the rhizome of *Coptis chinensis* Franch.; *C. deltoidea* C.Y.Cheng and P.K.Hsiao; *C. Teeta* Wall.), Pogostemonis Herba [from the whole plant *Pogostemon cablin* (Blanco). Benth], Sophorae Flavescentis Radix (from the root of *Sophora flavescens* Aiton), Atractylodis Rhizoma [from the rhizome of *Atractylodes lancea* (Thunb.) DC. or *A. chinensis* (Bunge) Koidz] and Crataegi Fructus (from the fruit of *Crataegus pinnatifida* Bunge. or *C. pinnatifida* var. *major* N.E.Br.) (Committee for the Pharmacopoeia of P.R. China, 2010; The Plant List, 2013), and the combination of these herbs clears heat, eliminates dampness, and acts as an astringent to stop diarrhea, invigorating the spleen to promote digestion, according to the theory of TCM (**Supplementary Material 1**).

In the clinic, Dihuang powder (DHP) has been shown to be effective for the treatment of diarrhea; however, until now, no studies of the toxicity and the anti-diarrheal activity of DHP have been performed with modern pharmacological technology. Therefore, it is necessary to evaluate its safety and treatment using standardized experimental protocols. In the present study, five compounds from the main medicinal materials were chosen to be biomarkers in a quality control evaluation, and the potential toxicity and the anti-diarrheal activity of DHP was investigated in a mouse model. Additionally, considering the relationship between diarrhea disease and abdominal pain as well as the inflammatory response of the body, we also studied the antinociceptive and anti-inflammatory activities of DHP. These tests provide a theoretical basis for the clinical use of DHP and may assist in the development of new drugs for the treatment of diarrhea.

## Materials and Methods

### Plant Material and Preparation of Dihuang Powder

Euphorbiae Humifusae Herba, Coptidis Rhizoma, Pogostemonis Herba, Sophorae Flavescentis Radix, Atractylodis Rhizoma and Crataegi Fructus were purchased at the Huanghe TCM market of Lanzhou City in Gansu Province, China in July of 2016 and were identified by Prof. Chaoying Luo, Lanzhou Institute of Husbandry and Pharmaceutical Sciences of Chinese Academy of Agricultural Sciences, China. All voucher specimens with accession numbers 20160101, 20160102, 20160103, 20160104, 20160105, and 20160106 were submitted to the Herbarium of Lanzhou Institute of Husbandry and Pharmaceutical Sciences, CAAS (Lanzhou, China). All medicinal materials for Dihuang powder were mixed according to the weight of each herb listed in **Table 1**. After pulverization with a grinder and filtering by a sieve, the powder was used in our tests at sizes that were as small as 120 meshes.

**Table 1:** The chemical component analysis of Dihuang powder.

	Quercetin	Berberine	Atractydin	Matrine	Patehouli aleohal
HPLC analysis	0.054 mg/g	0.632 mg/g	–^∗^	–	–
TLC analysis	–	–	+	+	+


*^∗^No determination.*

### Drugs and Reagents

Acetic acid (Tianjin Chemical Reagent Company, China); xylene (Shanghai Chemical Reagent Company, China); aspirin (Shenyang Aojina Pharmaceutical Company, China); diphenoxylate (Xinxiang Changle Pharmaceutical Company, China); castor oil (Shanghai Aladdin Industrial Corporation, China); and acetonitrile and methanol (HPLC grade) were purchased from Fisher Scientific (England); quercetin, berberine, atractylodin, matrine and patehouli aleohal were purchased from Shanghai Yuanye Biotechnology Co., Ltd. (Purity of all ≥98%, China).

### The Chemical Component Analysis of Dihuang Powder

In this test, five active compounds, including quercetin, berberine, atractylodin, matrine and patehouli aleohal from the main medicinal materials of DHP were chosen to be biomarkers in HPLC and TLC evaluation for the quality control.

#### High Performance Liquid Chromatography (HPLC) Analysis

**FIGURE 1:**
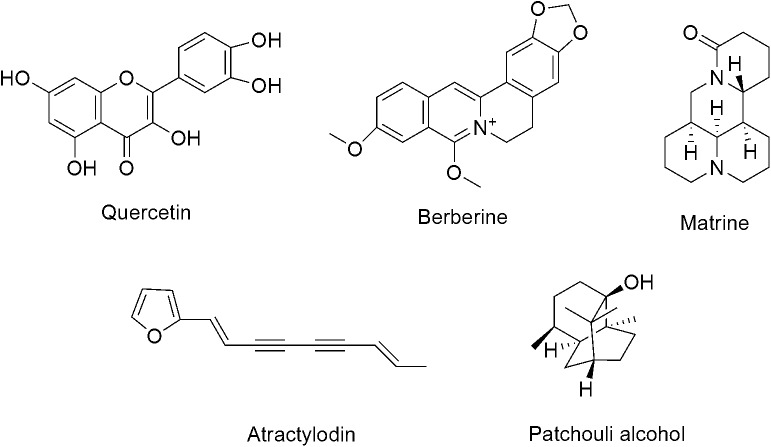
The chemical structure of five active compounds.

As the active compounds of Euphorbiae Humifusae Herba and Coptidis Rhizoma, the quercetin and berberine contents (**Figure 1**) in DHP were determined by HPLC (Committee for the Pharmacopoeia of P.R. China, 2010).

RP-HPLC analysis of DHP was performed on a Waters apparatus (two solvent delivery systems, model 600, and a Photodiode Array detector, model 996). The solvent system was composed of methanol (50%) and a 0.4% phosphoric acid solution (50%) for the identification of quercetin at 360 nm and acetonitrile (47%) and water (53%, contains potassium dihydrogen phosphate 3.4 g and sodium dodecylsulfate 1.7 g per 1000 ml) for the identification of berberine at 345 nm. Data acquisition and quantification were performed with millennium 2.10 version software (Waters). A symmetry reversed-phase column (250, 4.6, and 5 mm, Waters, Ireland) was maintained at ambient temperature (30.0°C). The mobile phase was filtered through a Millipore 0.45 mm filter and degassed prior to use. The peaks of quercetin and berberine were identified by comparison with chemical standards.

#### Thin Layer Chromatography (TLC) Analysis

In this test, as the active compounds of Atractylodis Rhizoma, Sophorae Flavescentis Radix and Pogostemonis Herba, atractylodin, matrine, and patehouli aleohal (**Figure 1**) in DHP were assayed by TLC according to the standardized experimental protocols of the Committee for the Pharmacopoeia of P.R. China, respectively (Committee for the Pharmacopoeia of P.R. China, 2010).

Petroleum ether (60–90°C)-acetone (9:2) was used as the developing solvent to develop atractylodin in the silica gel-CMC plate (Qingdao Haiyang Chemical Reagent Factory, China). Sulfuric acid (10%) with an ethanol solvent was used as the color developing reagent, and atractylodin was used as the standard preparation.

The below solution of chloroform-methanol-NH_3_^.^H_2_O (5:0.6:0.3) at 10°C was used as the developing solvent to develop matrine in the silica gel plate produced by 2% NaOH. Potassium iodide solution and sodium nitrites with ethanol solvent were used as the color developing reagents, and matrine was used as the standard preparation.

Petroleum ether (30–60°C)-acetic ether-acetic acid (95:5:0.2) was used as the developing solvent to develop patehouli aleohal in the silica gel-CMC plate. FeCl_3_ (5%) with an ethanol solvent was used as the color developing reagent, and patehouli aleohal was used as the standard preparation.

### Experimental Animals

Male or female Balb/C mice (18–22 g) were obtained from the Department of Animal Center, Lanzhou University (Lanzhou, China). They were kept in plastic cages at 22 ± 2°C with free access to pellet food and water. This study was carried out in accordance with the Regulation for the Administration of Affairs Concerning Experimental Animals (State Science and Technology Commission in China, 1988) and was approved by the Ministry of Health, P.R. China in accordance with the NIH guidelines (NIH, 2002), and the Ethics Committee of Lanzhou Institute of Husbandry and Pharmaceutical Sciences of the Chinese Academy of Agricultural Sciences (Lanzhou, China).

### Toxicity

#### Acute Toxicity

The up-and-down or staircase method for acute toxicity testing was carried out as previously described (Bruce, 1985). The dose was increased from 500 to 5000 mg/kg and was administered orally. Animals were observed continuously for behavioral changes for the first 4 h and then observed for mortality for 24 h after drug administration.

#### Sub-Acute Toxicity

In accordance with the GB15193.22-2014 Test Guidelines on 28-Day Repeated Dose Toxicity Study (China’s Ministry of Health, 2014), the OECD Guideline no. 407 (OECD, 1995) for principles of Good Laboratory Practices (FDA, 2010) and the described methods by Arbo et al. (2009), Akindele et al. (2014), Kong et al. (2016), and Peng et al. (2016) the test was carried out.

The animals were randomly divided into three treatment groups and a control group of 10 mice each (5 males and 5 females). DHP was dissolved in distilled water and administered orally for 28 consecutive days at the doses of 1000, 500, and 250 mg/kg, respectively, and the control group was orally administered the distilled water. Food and water were available *ad libitum* throughout the experiment. The behavioral changes, clinical signs of toxicity and mortality were observed and recorded, and the body weights of each mouse were measured at 7, 14, 21, and 28 days, respectively.

At the end of the treatment, the animals were fasted overnight of feed, but drinking water was available. Then, they were anesthetized, and the blood samples were collected. After euthanasia, target organs were collected for subsequent analysis.

Hematological parameters and biochemical parameters were assayed by an automatic hematology analyzer (BC-2800Vet, Mindray Co., China) and an automatic biochemistry analyzer (BS-420, Mindray Co., China), including white blood cells (WBC), lymphocytes (Lym), monocytes (Mon), red blood cells (RBC), hemoglobin concentration (HGB), hematocrit (HCT), mean corpuscular volume (MCV), mean corpuscular hemoglobin (MCH), mean corpuscular hemoglobin concentration (MCHC), platelets (PLT), and mean platelet volume (MPV); Glucose (GLU), creatinine (CRE), alkaline phosphatase (ALP), alanine aminotransferase (ALT), aspartate aminotransferase (AST), albumin (ALB), blood urea nitrogen (BUN), total protein (TP), total cholesterol (TCHO), and triglyceride (TG).

All the organs were examined and observed carefully, and the macroscopic pathological changes were recorded. The liver, spleen, kidney, stomach and small intestine were collected, weighed, and fixed in 10% buffered formalin and underwent a routine histological process for paraffin embedding and light microscopic examination, respectively. The relative organ weights were calculated based on the animal’s body weight.

### Anti-Diarrheal Activity

#### Effect of DHP on Sennae Induced Diarrhea

Anti-diarrheal activity was investigated according to the method described by Zavala-Mendoza et al. (2013), Wang et al. (2015), Wang et al. (2016), and Kinuthia et al. (2016), and Balb/C mice were randomly divided into six groups (10 animals per group). Three doses of DHP (1000, 500, and 250 mg/kg) were orally administered to each mouse; the model group received normal saline (10 ml/kg) and the positive group received diphenoxylate (5 mg/kg). After 30 min, a Sennae Folium decoction (1 g crude drug/ml, 0.2 ml/10 g weight, voucher specimen number 20160107) was administered to each mouse to induce diarrhea. Mice wasn’t administered to the decoction as control group.

Then, the animals were singly housed in separate cages lined with pre-weighed grease-proof paper, and the total mass of fecal output excreted after 6 h was measured every 2 h by subtracting the mass of the pre-weighed paper from the total mass of paper and fecal droppings.

#### Effect of DHP on Inflammatory Cytokines and Histopathology of Sennae Induced Diarrhea

The animals were randomly divided into five groups of 10 each and treated as the above assay. At the doses of 1000, 500, and 250 mg/kg, DHP were orally administered to each mouse, respectively; and the model group received normal saline and the positive group received diphenoxylate (5 mg/kg). After 30 min, a Sennae Folium decoction was administered to each mouse to induce diarrhea. Subsequently, they were anesthetized, and target organs were collected for subsequent analysis at 4 h. Small intestine was collected immediately and homogenized for 5 min with physiological saline in ice water. Then, the homogenates were transferred to a centrifuge tube and centrifuged at 2500 × g at 4°C for 10 min. The supernatant was collected and used to assay the activities of interleukin 1β (Il-1β), interleukin 6 (Il-6), and tumor necrosis factor-α (TNF-α) according to the manual of the Nanjing Jiancheng Elisa kit (Nanjing Jiancheng Bioengineering Institute, Nanjing, China). Three replicates were performed for each group.

Using the same test, the small intestine and large intestine were collected, weighed, and fixed in 10% buffered formalin and underwent a routine histological process for paraffin embedding and light microscopic examination, respectively.

#### Effect of DHP on Gastrointestinal Transit Time

This test was carried out according to the method described by Kinuthia et al. (2016). Briefly, 30 min after oral administration of DHP (1000, 500, and 250 mg/kg), normal saline (10 ml/kg), or diphenoxylate (5 mg/kg) to mice, a 10% suspension of charcoal meal (0.1 ml/10 g weight) was administered to each mouse by intragastric gavage. Thirty minutes later, mice were sacrificed by cervical dislocation, and the small intestine and stomach were immediately isolated. The distance traveled by the charcoal meal and total length of the intestine were measured, and the intestinal propulsion rate was expressed as the percentage of the distance traveled by the charcoal meal relative to the total length of the small intestine (Hu et al., 2009; Wang et al., 2015).

#### Effect of DHP on Castor Oil-Induced Diarrhea

Experiments were carried out according to a previously described method (Zavala-Mendoza et al., 2013). Thirty minutes after oral administration of treatments, diarrhea was induced via oral administration of castor oil (0.1 ml/10 g weight). Mice wasn’t administered to castor oil as control group. Animals were then singly housed in separate cages lined with pre-weighed grease-proof paper, and the total mass of fecal output excreted after 6 h was measured every 2 h by subtracting the mass of the pre-weighed paper from the total mass of the paper and fecal droppings.

### Antinociceptive Activity

The acetic acid-induced writhing response in mice was used to evaluate the antinociceptive activity of DHP. According to previously described methods (Shang et al., 2015b), 30 min after oral administration of DHP at doses of 1000, 500, and 250 mg/kg; normal saline (10 ml/kg) in the control group; and aspirin (100 mg/kg) in the positive group, 0.6% acetic acid (0.1 ml/10 g body weight) was intraperitoneally injected into mice. Mice were observed, and the number of abdominal constrictions and stretchings was determined after 15 min.

### Anti-Inflammatory Activity

Xylene-induced ear swelling in mice was used to investigate the anti-inflammatory activity. In accordance with a previously reported method (Shang et al., 2015b), DHP (1000, 500, and 250 mg/kg), normal saline (10 ml/kg), and aspirin (100 mg/kg) were orally administered to animals. Thirty minutes after the treatment, each animal received 30 μl of xylene on the anterior and posterior surfaces of the right ear lobe, and the left ear was considered the control. One hour later, the animals were sacrificed by cervical dislocation, and 8 mm diameter circular sections were taken from both ears with a cork borer and weighed. The degree of ear swelling was calculated based on the weight of the left ear that had been not been exposed to xylene.

### Statistical Analysis

The data were analyzed with the SPSS software program version 19.0 and are expressed as the mean ± SEM. Data were analyzed by one-way ANOVA, followed by Student’s two-tailed *t*-test for the comparison between the test and control group, and Dunnett’s test when the data involved three or more groups. *P*-values less than 0.05 (*P* < 0.05) were considered statistically significant.

## Results

### The Chemical Component Analysis of Dihuang Powder

**FIGURE 2:**
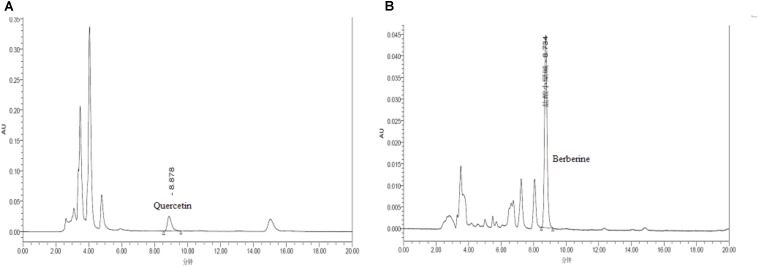
HPLC chromatogram for determining the content of quercetin **(A)** and berberine **(B)** in Dihuang powder.

**FIGURE 3:**
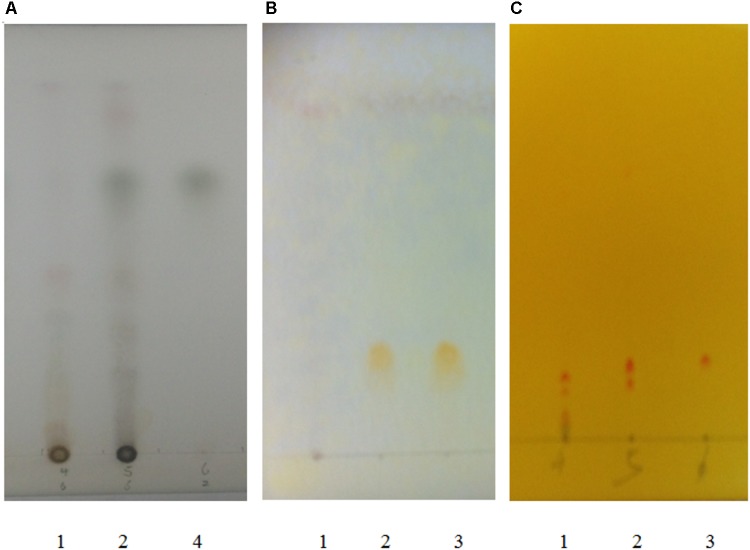
TLC chromatogram for detecting atractylodin **(A)**, patehouli aleohal **(B)**, and matrine **(C)** in Dihuang Powder. [**(A)** 1. The samples of DHP without Atractylodis Rhizoma, 2. The samples of DHP, and 3. Atractylodin; **(B)** 1. The samples of DHP without Pogostemonis Herba, 2. The samples of DHP, and 3. Patehouli aleohal; **(C)** 1. The samples of DHP without Flavescentis Radix, 2. The samples of DHP, and 3. Matrine].

Quercetin and berberine, the active compounds of Euphorbiae Humifusae Herba and Coptidis Rhizoma, were present in DHP at 0.054 and 0.632 mg/g, respectively, as per HPLC analysis (**Figure 2**). According to TLC analysis, atractylodin, matrine and patehouli aleohal were also present in DHP (**Table 1** and **Figure 3**).

### Acute Toxicity

Oral administration of DHP (500–5000 mg/kg) to mice did not cause death or acute behavioral changes during the observation periods, and no pathology changes were observed in mice. Therefore, the LD_50_ of DHP was estimated to be more than 5000 mg/kg by oral route.

### Sub-Acute Toxicity

#### The Body Weight and the Relative Organ Weights of Mice

After administrating orally DHP to mice for 28 days, the normal body weight gain was observed in both male and female mice, and no significant compared to control group (P > 0.05). Moreover, the relative organ weights of the treated and control groups also did not show any significant differences. During the experimental period, no any mortality or abnormal clinical signs of mice were presented (**Supplementary Material 2**).

#### Hematological and Serum Biochemical Parameters

From **Tables 2**, **3**, we can see that compared to the control group, some hematological and serum biochemical parameters were slight increased or decreased, such as the reduction of WBC for male mice and RBC, HCT, MCH, and MPV for female mice. But no significant differences (*P* > 0.05) were observed of three DHP treated-groups, and all of the change in these parameters were within the normal range of the testing laboratory (**Tables 2**, **3**).

**Table 2:** Effects of DHP on serum biochemical parameters of mice.

Parameter	Sex	Groups			
		Control	DHP		
			250 mg/kg	500 mg/kg	1000 mg/kg
WBC (×10^9^/L)	Male	5.25 ± 0.79	5.19 ± 0.82	5.09 ± 0.77	5.18 ± 0.63
	Female	4.98 ± 0.46	5.01 ± 0.87	4.86 ± 0.79	5.11 ± 0.91
Lym (%)	Male	76.76 ± 8.17	79.13 ± 7.04	72.43 ± 8.07	80.57 ± 7.64
	Female	75.23 ± 9.25	77.15 ± 8.25	69.89 ± 7.87	79.59 ± 6.13
Mon (%)	Male	4.85 ± 1.08	4.44 ± 1.24	4.64 ± 2.14	4.54 ± 1.81
	Female	4.76 ± 1.31	4.77 ± 1.15	4.22 ± 1.73	5.06 ± 1.56
RBC (×10^12^/L)	Male	12.39 ± 1.41	11.78 ± 1.54	12.08 ± 1.53	12.10 ± 0.84
	Female	12.45 ± 2.11	12.03 ± 1.81	11.91 ± 2.24	11.98 ± 3.10
HGB (g/L)	Male	158.11 ± 28.72	158.89 ± 25.55	153.14 ± 13.99	156.20 ± 23.19
	Female	161.12 ± 17.59	159.61 ± 31.23	156.54 ± 21.60	160.34 ± 27.42
HCT (%)	Male	65.59 ± 12.25	58.89 ± 9.14	60.97 ± 8.91	60.85 ± 5.21
	Female	55.39 ± 14.62	50.15 ± 10.81	53.27 ± 12.93	54.21 ± 9.94
MCV (fL)	Male	51.13 ± 1.72	50.54 ± 1.55	50.41 ± 1.66	50.30 ± 1.30
	Female	50.65 ± 2.71	49.88 ± 1.53	48.79 ± 2.12	51.01 ± 2.25
MCH (pg)	Male	15.55 ± 1.09	15.19 ± 0.85	14.82 ± 0.61	14.75 ± 0.21
	Female	16.16 ± 1.17	15.46 ± 0.86	15.51 ± 0.99	14.95 ± 0.87
MCHC (g/L)	Male	305.00 ± 16.63	302.75 ± 14.51	294.00 ± 4.36	297.00 ± 9.90
	Female	314.00 ± 15.57	307.00 ± 9.91	311.25 ± 8.78	305.00 ± 10.10
PLT (×10^9^/L)	Male	1003.25 ± 238.79	910.01 ± 207.11	1109.38 ± 154.69	946.11 ± 154.47
	Female	859.19 ± 223.31	781.89 ± 131.34	884.45 ± 116.76	798.34 ± 165.83
MPV (fL)	Male	5.91 ± 0.41	5.84 ± 0.60	5.18 ± 0.33	5.44 ± 0.54
	Female	6.01 ± 0.58	5.65 ± 0.46	5.78 ± 0.74	5.85 ± 0.49


**Table 3:** Effects of DHP on serum biochemical parameters of mice.

Parameter	Sex	Groups			
		Control	DHP		
			250 mg/kg	500 mg/kg	1000 mg/kg
GLU (mmol/L)	Male	3.14 ± 0.48	3.03 ± 0.16	3.13 ± 0.34	2.91 ± 0.28
	Female	2.98 ± 0.38	2.94 ± 0.78	3.24 ± 0.54	3.10 ± 0.44
CRE (μmol/L)	Male	24.50 ± 2.17	19.75 ± 7.43	20.86 ± 4.37	20.57 ± 2.64
	Female	25.23 ± 4.25	20.15 ± 4.25	19.89 ± 3.87	19.59 ± 4.13
ALP (U/L)	Male	132 ± 10.08	129.11 ± 8.46	126.83 ± 21.11	137.52 ± 8.81
	Female	157.76 ± 12.09	147.69 ± 10.50	142.16 ± 33.94	150.61 ± 16.20
ALT (U/L)	Male	65.62 ± 7.39	65.26 ± 3.66	67.86 ± 11.32	59.70 ± 13.02
	Female	55.34 ± 5.78	51.23 ± 4.56	53.29 ± 8.87	50. 34 ± 11.04
AST (U/L)	Male	152.87 ± 24.82	168.33 ± 13.29	153.98 ± 19.76	161.99 ± 34.66
	Female	140.11 ± 10.23	155.73 ± 11.67	151.23 ± 8.97	148.54 ± 21.23
ALB (g/L)	Male	32.99 ± 4.16	31.08 ± 6.96	28.08 ± 3.51	29.64 ± 3.16
	Female	30.13 ± 5.73	29.87 ± 5.88	28.11 ± 2.78	28.88 ± 4.91
BUN (mmol/L)	Male	10. 45 ± 1.36	8.38 ± 3.48	8.63 ± 2.80	8.92 ± 2.81
	Female	10.75 ± 3.54	9.10 ± 4.15	8.99 ± 3.64	9.10 ± 4.82
TP (g/L)	Male	57.59 ± 4.30	57.22 ± 6.12	52.40 ± 5.27	53.32 ± 8.15
	Female	59.65 ± 8.67	60.15 ± 5.89	58.78 ± 9.10	58.42 ± 8.79
TCHO (mmol/L)	Male	3.10 ± 0.27	3.18 ± 1.04	2.84 ± 0.38	2.80 ± 0.17
	Female	2.78 ± 0.34	2.83 ± 0.87	2.71 ± 0.45	2.66 ± 0.34
TG (mmol/L)	Male	1.52 ± 0.33	1.54 ± 0.22	1.74 ± 0.24	1.76 ± 0.37
	Female	1.34 ± 0.34	1.29 ± 0.19	1.45 ± 0.42	1.49 ± 0.19


#### Histopathological Observation

**FIGURE 4:**
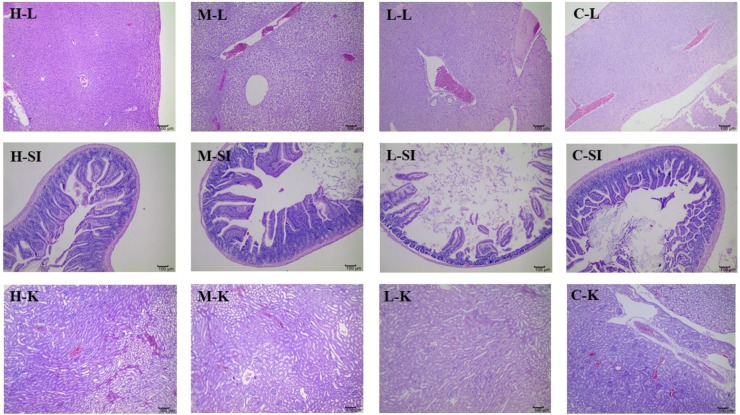
Microphotographs of liver (L), kidney (K), and small intestine (SI) at 100× of mice in three DHP-treated groups (low dose, L; middle dose, M; high dose, H), and control group (C).

**FIGURE 5:**
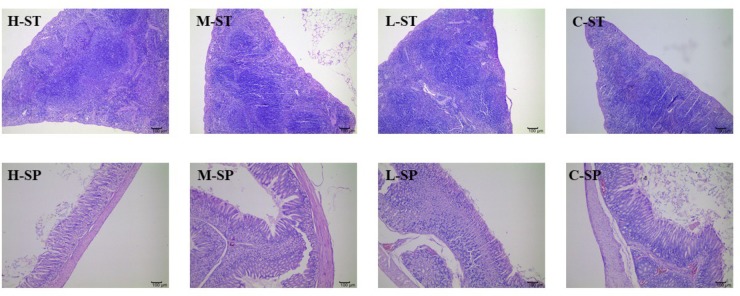
Microphotographs of spleen (SP) and stomach (ST) at 100× of mice in three DHP-treated groups (low dose, L; middle dose, M; high dose, H) and control group (C).

**Figures 4**, **5** presented the microphotographs of liver, spleen, kidney, stomach and small intestine of mice in three DHP-treated groups and control group, respectively. Results showed that after administrating DHP to mice for 28 consecutive days, compared to the control group, no histopathological changes were observed in the histological examination of the organ sections in the tissue of mice (**Figures 4**, **5**).

### Anti-diarrheal Activity

#### Effect of DHP on Sennae Induced Diarrhea

As shown in **Table 4**, compared to the control group (4.56 ± 0.48 g), DHP at doses of 1000 mg/kg (1.90 ± 0.41 g, P < 0.01), 500 mg/kg (2.81 ± 0.41 g, P < 0.01), and 250 mg/kg (4.26 ± 0.37 g, P < 0.01) decreased the weight of feces excreted from mice in a dose-dependent manner, and inhibition of diarrhea was 58.23, 38.34, and 6.58% for 1000, 500, and 250 mg/kg, respectively. The positive drug diphenoxylate significantly reduced the weight of mice, with an inhibition of 63.38% (**Table 4**).

**Table 4:** Effect of Dihuang powder on Sennae induced diarrhea.

Groups	Dose (mg/kg)	Weight of fecal output excreted (g)				Inhibition (%)
		0 ~ 2 h	2 ~ 4 h	4 ~ 6 h	Total	
DHP	1000	0.46 ± 0.25^∗∗^	1.09 ± 0.38^∗∗^	0.26 ± 0.21	1.90 ± 0.41^∗∗^	58.34
	500	1.34 ± 0.46^∗∗^	1.19 ± 0.52^∗∗^	0.29 ± 0.12	2.81 ± 0.41^∗∗^	38.34
	250	2.04 ± 0.54	1.93 ± 0.42	0.29 ± 0.16	4.26 ± 0.37	6.58
Diphenoxylate	5	0.41 ± 0.70^∗∗^	0.91 ± 0.20^∗∗^	0.27 ± 0.28	1.67 ± 0.22^∗∗^	63.38
Model	–	2.06 ± 0.54	2.23 ± 0.55	0.23 ± 0.13	4.56 ± 0.48	–
Control	–	0.44 ± 0.12	0.06 ± 0.05	0.10 ± 0.06	0.60 ± 0.16	–


*Each value represents the mean ± SEM of 10 mice.*

*^∗^P < 0.05 compared with model group. ^∗∗^P < 0.01 compared with model group.*

#### Effect of DHP on Inflammatory Cytokines and Histopathology of Sennae Induced Diarrhea

**FIGURE 6:**
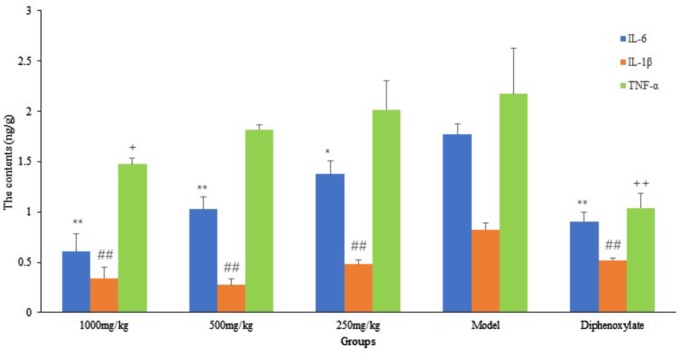
The contents of inflammatory cytokines in small intestine. ^∗^*P* < 0.05 compared with model group, ^∗∗^*P* < 0.01 compared with model group for IL-6; ^#^ *P* < 0.05 compared with model group, ^##^*P* < 0.01 compared with model group for IL-1β; *^+^P* < 0.05 compared with model group, *^++^P* < 0.01 compared with model group for TNF-α.

From **Figure 6**, we can see that compared with the model group, the contents of IL-6 in the small intestine of DHP groups (1000 and 500 mg/kg) were decreased significantly (*P* < 0.01), and the content of IL-1β in all the DHP treatment groups were significantly lower than model group (*P* < 0.01). But for TNF-α, only high dose group (1000 mg/kg) was significant lower than that in the model group (*P* < 0.05), but there was no significant difference in other two groups (*P* > 0.05). As the positive drug, diphenoxylate decreased significantly the contents of IL-6, IL-1β, and TNF-α in the small intestine (*P* < 0.01).

**FIGURE 7:**
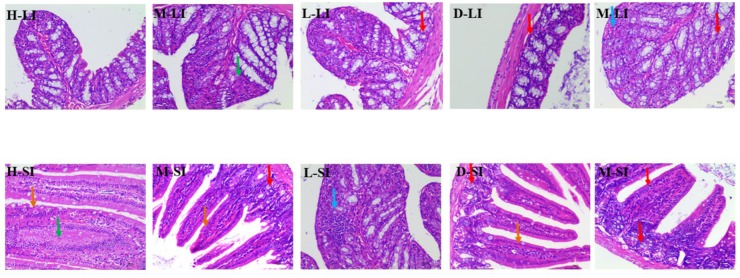
Histopathological changes of small intestine and large intestine. LI, large intestine; SI, small intestine; arrows indicate the injuries. Original magnification, ×400.

In the Sennae induced diarrhea mice, the integrity of the intestinal lamina propria cells was destroyed, the epithelial cell of intestines mucous proliferation and the inflammatory cellular infiltrations and congestion were observed. Meanwhile, in large intestine, the glandular vacuolar degeneration was appeared. After the treatment with DHP and diphenoxylate, the above histopathological changes were effectively ameliorated or completely eliminated (**Figure 7**).

#### Effect of DHP on Gastrointestinal Transit

Results showed that 30 min after administration of a charcoal meal, DHP reduced the distance traveled by the charcoal meal in mice in a dose-dependent manner. The 1000 mg/kg dose of DHP reduced the rate to 74.22% compared to the control group (88.85%) (P < 0.05). Diphenoxylate also significantly decreased the movement rate by 67.07% (P < 0.01) (**Table 5**).

**Table 5:** Effect of Dihuang powder on gastrointestinal transit.

Groups	Dose (mg/kg)	Mean length of small intestine (cm)	Mean distance traveled by charcoal meal (cm)	Mean% movement of charcoal meal
DHP	1000	43.45 ± 2.64	32.28 ± 4.82	74.22 ± 9.91^∗^
	500	44.33 ± 2.87	33.55 ± 4.78	75.78 ± 10.15
	250	42.44 ± 0.05	34.56 ± 4.88	81.87 ± 8.78
Diphenoxylate	5	43.35 ± 2.78	29.08 ± 4.97	67.07 ± 10.47^∗∗^
Control	-	44.93 ± 2.30	39.83 ± 2.03	88.86 ± 6.45


*Each value represents the mean ± SEM of 10 mice.*

*^∗^P < 0.05 compared with control. ^∗∗^P < 0.01 compared with control.*

#### Effect of DHP on Castor Oil-Induced Diarrhea

In addition to Sennae Folium, castor oil was also used to induce diarrhea. The results showed that DHP exerted a strong effect and significantly decreased the weight of fecal matter excreted by mice induced by castor oil within 4 h. At doses of 1000, 500, and 250 mg/kg, the fecal weights in the DHP treated group were 1.69, 2.84, and 3.06 g, respectively, and the weight in the positive group was 1.54 g. Compared to the control group, the inhibition rates of the above four groups were 56.00, 26.65, 20.32, and 59.90%, respectively (**Table 6**).

**Table 6:** Effect of Dihuang powder on castor oil induced diarrhea.

Groups	Dose (mg/kg)	Weight of fecal output excreted (g)				Inhibition (%)
		0 ~ 2 h	2 ~ 4 h	4 ~ 6 h	Total	
DHP	1000	0.46 ± 0.25^∗∗^	0.91 ± 0.44^∗∗^	0.31 ± 0.12	1.69 ± 0.43^∗∗^	56.00
	500	0.77 ± 0.29^∗^	1.79 ± 0.21^∗∗^	0.29 ± 0.12	2.84 ± 0.49^∗^	26.05
	250	1.46 ± 0.33	1.31 ± 0.35	0.29 ± 0.16	3.06 ± 0.66	20.32
Diphenoxylate	5	0.37 ± 0.14^∗∗^	0.84 ± 0.26^∗∗^	0.22 ± 0.11	1.54 ± 0.29^∗∗^	59.90
Model	–	1.29 ± 0.22	2.27 ± 0.39	0.29 ± 0.18	3.84 ± 0.39	–
Control	–	0.44 ± 0.12	0.06 ± 0.05	0.10 ± 0.06	0.60 ± 0.16	–


*Each value represents the mean ± SEM of 10 mice.*

*^∗^P < 0.05 compared with model group. ^∗∗^P < 0.01 compared with model group.*

### Antinociceptive Activity

As shown in **Table 6**, DHP exhibited antinociceptive activity in the acetic acid-induced writhing response test in a dose-dependent manner. Compared to the control group (11.00 ± 1.60), DHP at 1000 mg/kg could significantly decrease the number of writhings (3.88 ± 0.99, *P* < 0.01), and its inhibition rate was 64.73%. Aspirin also showed antinociceptive activity, with an inhibition rate of 85.19% (**Table 7**).

**Table 7:** Antinociceptive activity of Dihuang powder.

Groups	Dose (mg/kg)	Number of writhings	Inhibition (%)
DHP	1000	3.88 ± 0.99^∗∗^	64.73%
	500	6.63 ± 1.51^∗∗^	39.73%
	250	9.75 ± 1.98	11.37%
Aspirin	100	1.63 ± 0.74^∗∗^	85.19%
Control	–	11.00 ± 1.60	–


*Each value represents the mean ± SEM of 10 mice.*

*^∗^P < 0.05 compared with control. ^∗∗^P < 0.01 compared with control.*

### Anti-inflammatory Activity

**Table 7** shows that DHP at doses of 1000, 500, and 250 mg/kg dose-dependently suppressed xylene induced ear swelling in mice by 39.81% (*P* < 0.01), 20.95 and 10.48%, respectively. Aspirin (100 mg/kg) showed marked anti-inflammatory activity and has a 36.71% reduction compared to the control group (*P* < 0.01) (**Table 8**).

**Table 8:** Anti-inflammatory activity of Dihuang powder.

Groups	Dose (mg/kg)	Swelling (%)	Inhibition (%)
DHP	1000	51.79 ± 10.61^∗∗^	39.81
	500	68.20 ± 10.23	20.95
	250	77.03 ± 10.70	10.48
Aspirin	100	54.46 ± 12.87^∗∗^	36.71
Control	–	86.04 ± 9.59	–


*Each value represents the mean ± SEM of 10 mice.*

*^∗^P < 0.05 compared with control. ^∗∗^P < 0.01 compared with control.*

## Discussion

To restore personal comfort and convenience, many patients require anti-diarrheal therapy and treatment is carried out to achieve (Bello et al., 2016). Dihuang powder is used as folk formulation for treating diarrhea, and is composed of six herbal medicines. The principal drug Euphorbiae Humifusae Herba clears away heat-evil, expels superficial evils, and cools blood. The drugs Coptidis Rhizoma and Pogostemonis Herba can clear heat, eliminate dampness, and purge fire to remove toxins. Other drugs, including Sophorae Flavescentis Radix, Atractylodis Rhizoma, and Crataegi Fructus, were used as adjuvant drugs to promote digestion, invigorate the stomach, promote Qi and eliminate stasis to activate blood circulation. Modern pharmacological studies also revealed various significant bioactivities of the above herbs and their active compounds (quercetin, berberine, matrine, etc.), including anti-diarrheal, antibacterial, antifungal, antioxidant, anti-inflammatory, antiviral and analgesic activities (Yi et al., 2013; Luyen et al., 2014), and they may contribute to the anti-diarrheal activity of DHP. For example, as the main principle of DHP, Coptidis Rhizoma (*Huanglian*) and its active compound berberine with the significant antimicrobial, antiprotozoal, antidiarrheal, and antitrachoma and other activities, have been made to anti-diarrheal agents, and widely used to treat diarrhea and dysentery in China (Committee for the Pharmacopoeia of P.R. China, 2010; Kumar et al., 2015; China Food and Drug Administration, 2017). Therefore, control of the quality of medicinal materials and preparations with modern analytical tools is very important to ensure their efficacy. In this study, two active compounds of Euphorbiae Humifusae Herba and Coptidis Rhizoma in DHP were identified using HPLC, and three compounds from other herbs were assayed. The results showed that the contents of quercetin and berberine were 0.054 and 0.632 mg/g, respectively, and atractylodin, matrine and patehouli aleohal were also found in DHP. We thought that the quality of DHP was good under this condition.

In addition, considering the importance of the toxicity of herbal medicines, acute toxicity was investigated. The results of acute oral toxicity showed that the LD_50_ of DHP was estimated to be more than 5000 mg/kg. According to the guide of OECD (2002a,b), the extracts would be considered relatively safe when the acute oral LD_50_ value of more than 3000–5000 mg/kg, we thought DHP is safe on acute oral toxicity. Then, the sub-acute test was carried out to further evaluate toxicity. After administrating DHP to mice for 28 days, no significant changes were observed in the general behavior and fur color in all of the DHP-treated groups, and the body weight and the relative organ weight of mice also didn’t be significantly changed compared to control group. Moreover, at the end of the experiment, all tested hematological and serum biochemical parameters were within the normal range, and no significant differences were observed, which were considered as the sensitive indicators of the toxicity of drugs and chemicals, they also give an important index for physiological and pathological status in humans and animals in general (Olson et al., 2000). Meanwhile, because the liver and kidney are sensitive organs, and their functions are known to be affected by a number of factors, such as drugs, ultimately leading to renal failure and liver toxicity (Li et al., 2016), and stomach and small intestine are main responsible for digesting the food or drugs administrated to mice. In our assay, the histopathological changes of liver, spleen, kidney, stomach, and small intestine of mice in three DHP-treated groups and control group also were investigated, respectively. Compared to the control group, no toxicological or pathological changes were caused, and DHP didn’t change the histopathological morphology of five organs in mice after 28 consecutive days. Based on the above results, we thought that DHP is non-toxic at all the tested dosages to animal, and did not develop any signs of toxicity or any evidence of systems disruptions.

As a TCM, Sennae Folium (the leaves of *Cassia angustifolia* Vahi or *C. angustifolia* Delile) purges heat, relieves diuresis and is widely used as a cathartic in China. It stimulates peristalsis of the small intestine and colon and releases inflammatory factors to increase the permeability of the small intestine epithelium (Zhang et al., 2014). Additionally, castor oil could cause water and electrolyte permeability changes in the intestinal mucosal membranes, resulting in fluid and watery luminal content that flow rapidly through the small and large intestines (Gaginella et al., 1975; Mbagwu and Adeyemi, 2008; Awe et al., 2011), and through elevated the production of several mediator substances that include prostaglandins, nitric oxide, and platelet activating factor, cAMP and tachykinins (Izzo et al., 1999; Besra et al., 2002; Brijesh et al., 2009). Therefore, in our study, Sennae and castor oil were used to induce diarrhea in the experimental animals. The results showed that DHP decreased the total weight of fecal matter excreted by mice induced by Sennae and castor oil in a dose-dependent manner, and 1000 and 500 mg/kg doses exhibited significant anti-diarrheal activity within 2 and 4 h (*P* < 0.01, **Tables 4**, **6**). DHP also could decrease the content of IL-1β, IL-6, and TNF-α in the small intestine induced by Sennae, and improve the histopathological changes of small intestine and large intestine of model group (**Figures 6**, **7**). Additionally, DHP reduced the mean distance traveled by the charcoal meal, and the high-dose drug was associated with 74.22% movement of charcoal meal (*P* < 0.05) compared to the control group (88.86%) (**Table 5**). These tests indicated that DHP has good anti-diarrheal activity by reducing gastrointestinal motility and secretions, and the elicited effects were similar to those many anti-diarrheal agents (Gutiérrez et al., 2013). Meanwhile, DHP may possess the anti-inflammatory activity by inhibiting prostaglandin biosynthesis.

Because the decrease of the inflammatory response and alleviation of pain all are very important for the treatment of this disease in the clinic, the acetic acid-induced writhing response in mice and xylene-induced ear swelling in mice were used to evaluate the possible antinociceptive and anti-inflammatory activities. Acetic acid produced a painful reaction and acute inflammation in the peritoneal area, which was considered to be a non-selective antinociceptive model. Xylene-induced neurogenous swelling partially was associated with substance P, as a common inflammatory model for evaluating vascular permeability (Shang et al., 2011). Results demonstrated that DHP decreased the number of writhings induced by acetic acid and decreased the ear swelling in mice induced by xylene in a dose-dependent manner (**Tables 7**, **8**). Therefore, we thought that DHP could help decrease the inflammatory response and alleviate pain when treating diarrhea.

## Conclusion

In this study, Dihuang powder is safe at the given doses in terms of acute and sub-acute toxicity. It exhibits anti-diarrheal, antinociceptive and anti-inflammatory activities as well as reduced gastrointestinal motility and the secretions induced by Sennae and castor oil. It also decreased the inflammatory response and alleviated pain. These results provide evidence for the traditional uses of DHP and indicate that it can be used to treat diarrhea. The comprehensive mechanism of the anti-diarrheal activity of DHP should be studied further.

## Author Contributions

XS and XM conceived the paper. XS, XG, BL, and FY carried out the tests and wrote the manuscript. HP and JZ collected and analyzed the literature. YZ edited the manuscript. All authors read and approved the final version of the manuscript.

## Conflict of Interest Statement

The authors declare that the research was conducted in the absence of any commercial or financial relationships that could be construed as a potential conflict of interest.

## Abbreviations

DHP: Dihuang powder
HPLC: high performance liquid chromatography
LD_50_: median lethal dose
TCM: traditional Chinese medicine
TLC: thin layer chromatography
WHO: World Health Organization
