# Supplementary material for: The Anti-diarrheal Activity of the Non-toxic Dihuang Powder in Mice
Source: Front Pharmacol. 2018 Sep 13;9:1037. doi: 10.3389/fphar.2018.01037 (PMC6147127; doi:10.3389/fphar.2018.01037)
Supplement: Supplementary file 1 [file Table_1.DOCX]

The anti-diarrheal activity of the nontoxic Dihuang powder in mice

Xiaofei Shang^a^, Xiaolou Miao^a^, Feng Yang^a^, Bing Lia, Xiao Guo^b*^, Hu Pan^a^, Yu Zhang^c*^, Jiyu Zhang^a*^

*^a^ Key Laboratory of New Animal Drug Project, Gansu Province, Key Laboratory of Veterinary Pharmaceutical Development of Ministry of Agriculture, Lanzhou Institute of Husbandry and Pharmaceutical Sciences of Chinese Academy of Agricultural Science, Lanzhou 730050, PR China*

*^b^ Tibetan Medicine Research Center of Qinghai University, Qinghai University Tibetan Medical College, Qinghai University, Xining 810016, PR China*

*^c^ Department of Emergency, Lanzhou Army General Hospital, Lanzhou 730050, PR China*

*Corresponding author; Tel.: +86-931-2115262

E-mail address: shangxf928@126.com

Supplementary material 1. Composition of Dihuang powder

| Pharmaceutical names | Plant names | Family | Chinese  name | Traditional uses* | Weight |
| --- | --- | --- | --- | --- | --- |
| Euphorbiae Humifusae Herba | *Euphorbia humifusa* Willd.;  *E. maculata* L. | *Euphorbiaceae* | Dijincao | Clearing away the heat-evil and expelling superficial evils, cooling blood to stop bleeding, and eliminating dampness. | 35% |
| Coptidis Rhizoma | *Coptis chinensis* Franch.;  *C. deltoidea* C.Y.Cheng et Hsiao  *C. teeta* wall. | *Ranunculaceae* | Huanglian | Clearing heat and eliminating dampness, and purging fire to remove toxins. | 20% |
| Pogostemonis Herba | *Pogostemon cablin* (Blanco) Benth. | *Labiatae* | Guang Huoxiang | Eliminating wetness-evil with drugs of fragrant flavor, regulating the middle warmer and stopping vomit, relieving summer-heat. | 20% |
| Sophorae Flavescentis Radix | *Sophora flavescens* Ait. | *Leguminosae* | Kushen | Clearing heat and eliminating dampness, insect disinfestation and diuresis. | 10% |
| Atractylodis Rhizoma | *Atractylodes lancea* (Thunb.) DC.;  *A. chinensis* (DC.) Koidz. | *Asteraceae* | Cangzhu | Eliminating dampness and strengthening the spleen, eliminating dampness. | 10% |
| Crataegi Fructus | *Crataegus pinnatifida* Bge.;  *C. pinnatifida* Bge. var. *major* N.E.Br. | *Rosaceae* | Shanzha | Promoting of digestion and invigorating stomach, promoting qi and eliminating stasis to activate blood circulation. | 5% |

* Information about medicinal materials was obtained from the Committee for the Pharmacopoeia of P.R. China (2010).

Supplementary material 2. Effects of DHP on the body weights and the relative organ weights (%) of mice

| Group | Dose  (mg/kg) | Sex | Initial body  weight (g) | Final body  Weight (g) | Liver  (%) | Stomach  (%) | Spleen  (%) | Kidneys  (%) | Small intestine (%) |
| --- | --- | --- | --- | --- | --- | --- | --- | --- | --- |
| DHP | 1000 | Male | 22.57 ± 1.60 | 32.79 ± 2.76 | 4.92 ± 0.45 | 2.37 ± 0.37 | 0.23 ± 0.03 | 1.26 ± 0.20 | 6.60 ± 1.04 |
|  |  | Female | 21.34 ± 1.37 | 30.87 ± 2.78 | 4.75 ± 0.36 | 2.21 ± 0.25 | 0.21 ± 0.03 | 1.18 ± 0.16 | 6.24 ± 0.87 |
|  | 500 | Male | 22.51 ± 1.09 | 35.40 ± 3.68 | 4.69 ± 0.78 | 2.12 ± 0.58 | 0.21 ± 0.07 | 1.32 ± 0.14 | 6.30 ± 1.01 |
|  |  | Female | 20.96 ± 1.15 | 29.98 ± 2.36 | 4.53 ± 0.53 | 2.22 ± 0.45 | 0.21 ± 0.03 | 1.22 ± 0.24 | 6.11 ± 0.79 |
|  | 250 | Male | 22.63 ± 1.39 | 33.32 ± 2.49 | 5.04 ± 0.93 | 2.03 ± 0.41 | 0.21 ± 0.03 | 1.35 ± 0.16 | 6.44 ± 0.51 |
|  |  | Female | 22.12 ± 1.52 | 32.15 ± 2.39 | 4.89 ± 0.66 | 2.05 ± 0.32 | 0.20 ± 0.04 | 1.24 ± 0.23 | 6.38 ± 0.97 |
| Control | -- | Male | 22.59 ± 1.49 | 33.97 ± 3.13 | 4.89 ± 0.74 | 2.16 ± 0.31 | 0.18 ± 0.06 | 1.30 ± 0.14 | 6.23 ± 0.96 |
|  |  | Female | 21.58 ± 1.42 | 31.09 ± 3.11 | 4.72 ± 0.63 | 2.06 ± 0.49 | 0.19 ± 0.05 | 1.24 ± 0.21 | 6.14 ± 0.45 |
